# Decision-making at the limit of viability: the Austrian neonatal choice context

**DOI:** 10.1186/s12887-019-1569-5

**Published:** 2019-06-20

**Authors:** Michal Stanak, Katharina Hawlik

**Affiliations:** 1 Ludwig Boltzmann Institute for Health Technology Assessment, Garnisongasse 7/20, 1090 Vienna, Austria; 2 Department of Philosophy, University of Vienna, Vienna, Austria

**Keywords:** Neonatology, NICU, Limit of viability, Decision-making, Choice context, Communication strategies

## Abstract

**Background:**

We aimed to explore the shared decision-making context at the *limit of viability* (weeks 22–25 of gestation) through analyzing neonatologist’s communication strategies with parents and their possible impact on survival and neurodevelopmental impairment (NDI) outcomes.

**Methods:**

A mixed methods approach was applied where a systematic literature search and in-depth semi-structured interviews with five heads of neonatology departments and one clinical ethicist from the Austrian context were integrated into a literature review. The aim was to identify decision practice models and the choice context specific to Austria.

**Results:**

Professional biases, parental understanding, and the process of information giving were identified as aspects possibly influencing survival and NDI outcomes. Institutions create self-fulfilling prophecies by recommending intensive/palliative care based upon their institutional statistics, yet those vary considerably among high-income countries. Labelling an extremely preterm (EP) infant by the gestational week was shown to skew the estimates for survival while the process of information giving was shown to be subject to framing effect and other cognitive biases.

**Conclusion:**

Communication strategies of choice options to parents may have an impact on the way parents decide and hence also on the outcomes of EP infants.

## Background

Globally, less than 1 % of all pregnant women give birth extremely preterm (EP), before the completion of 28 weeks of pregnancy [1]. In Austria, 350 infants were born extremely preterm in 2016, accounting for 0.4% of all births [2]. Despite these relatively small numbers, extreme prematurity is the leading cause of infant death [3]. It is also potentially related to short and long-term morbidity accounting for almost 45% of children with cerebral palsy, 35% with visual impairment, and 25% with cognitive or hearing impairment in the US [4].

The ways in which EP births are currently managed include prevention, preparation for the delivery, as well as intensive and palliative (comfort) care treatment options post-delivery. It is particularly between weeks 22 to 25 of gestation - the *limit of viability,* when shared decision-making with parents concerning intensive and comfort care options is at stake. The intensive care options include the application of surfactant therapy, intubation, and supportive ventilation, while comfort care options aim at improving an infant’s quality of life (QoL), treating symptoms, and minimizing pain and suffering [5].

*Limit of viability* is the point in foetal development at which the EP infant has a chance for extra-uterine survival [6]. This definition of the *limit of viability* is changing over time due to improvements in treatment and care. These improvements lead also to better survival and neurodevelopmental impairment (NDI) outcomes that differ between institutions and countries [7]. However, there is a considerable consensus among high income countries that with intensive care, most infants born after 25 weeks and 0 days (25 + 0) of gestational age (GA) will survive, while there is a little chance for survival and survival without severe impairment in infants born below 22 + 0 weeks of GA [6]. The probability of survival and survival without impairment increases significantly over these few weeks (22 + 0 to 25 + 6) [8]. In the context of intensive and comfort care, determining this point with as much precision as possible is important in order to prevent inflicting unnecessary burden on the infant and the family on the one hand, yet to give sufficient chances for survival to the infant on the other hand.

Finding the agreement during shared decision-making with parents of EP infants before and after the delivery is one of the key tasks of the Neonatal Intensive Care Unit (NICU) team. This decision-making is occurring at the backdrop of a specific cultural, socio-economic, and religious context and because the content that needs to be communicated is highly sensitive, it places even more emphasis on the form of communication. However, neonatologist’s communication strategies with parents in preparation for delivery as well as after delivery are not addressed in current guidelines (GLs). The fact that the form of communication has a significant impact on the person’s decision-making is well supported by research from behavioural sciences [9] and hence it is assumed that the same applies to the NICU context of choice. The design in which the options of choices are communicated is assumed to have an impact on the way parents decide and hence on the survival and NDI outcomes of EP infants.

In this review, we aimed to explore the shared decision-making context through reviewing the general literature and comparing it to the specific Austrian context in which we conducted qualitative interviews with the heads of NICUs and a clinical ethicist. This paper is a shortened version of one part of a larger health technology assessment (HTA), which particularly focuses on the analysis of the neonatologist’s communication strategies with parents and their possible impact on survival and NDI outcomes of EP infants. The HTA was conducted to provide decision support for resource planning of NICUs in Austria and additionally included the assessment of outcomes, resources, and ethical challenges [10, 11].

## Methods

A mixed methods approach was applied. In the first step, a systematic literature search was conducted to identify the most relevant sources applying the MIP (Methodology, Issue, Participants) question and inclusion criteria as listed in the Table 1. Secondly, interviews with the heads of departments of neonatology of five perinatal care centres and a clinical ethicist working at a NICU were conducted to gather data specific to the Austrian neonatal context. Data from the systematic search as well as the interviews were integrated into a literature review. Each part of the text thus includes data from the literature review that reflects the general state of the neonatal literature and supports it by the context specific data from interviews (presented in the running text or standing alone as quotes).

**Table 1:** Methodology, Issue, Participants (MIP), and inclusion criteria for systematic search

Methodology	Include empirical studies. Both quantitative and qualitative studies – surveys, in-depth interviews, questionnaires, etc.
Issue	Limit of viability, threshold of viability, border of viability, children born at 22 to 25 week of gestation, extremely preterm birth, gestational age 22 + 0 to 25 + 6, end of life treatment, “best practice”/“good practice” models of decision-making, social factors, ethics, ethical/moral challenges/dilemmas
Participants	parents, doctors (physicians), ethical council, ethical committee
Setting	Neo-natal intensive care units (NICU)
Publication period	1990–2017
Languages	German/English

### Systematic literature search

The systematic literature search was conducted in the period between 21.06.2017 and 23.06.2017 in the following databases:

- Medline via Ovid
- Embase
- The Cochrane Library
- CRD (DARE, NHS-EED, HTA)
- PsychInfo
- CINAHL

**Fig. 1:**
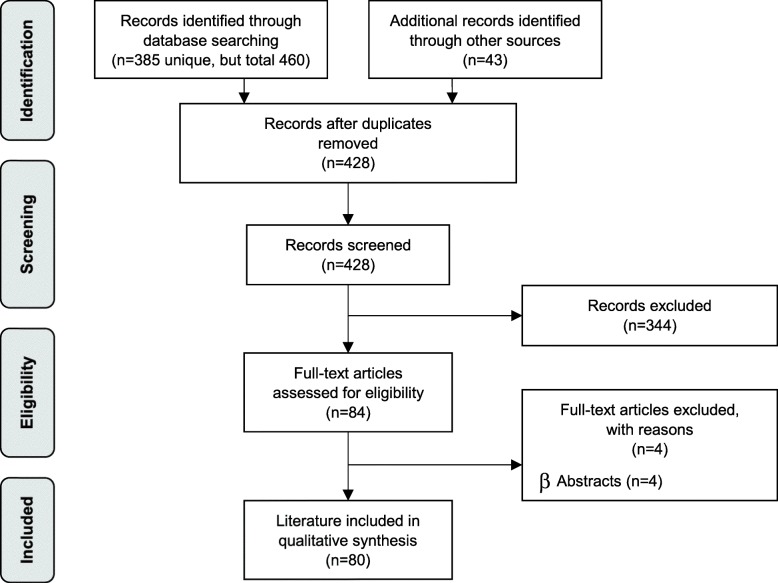
Flow chart of study selection (PRISMA Flow Diagram)

The systematic search was not limited to a specific study design, but it was limited to specific languages (German and English) and the publication period 1990–2017. The reason for limiting the year of publication to 1990 was to limit the volume of hits and to focus on the most up-to-date decision models in neonatal care. After deduplication, overall 385 citations were included. The specific search strategy employed can be found in the availability of data and materials section. In addition, a hand search of literature (web-search) was performed and yielded additional 43 sources, resulting in overall 428 hits. 80 publications were finally selected for the analysis (see Fig. 1: PRISMA Flow Diagram).

The data retrieved from the 80 publications selected for the analysis were not extracted into extraction tables, but were analysed according to content analysis where all the topics relevant to the theme of biases in neonatal decision-making were included. In terms of literature selection, one author (MS), reviewed the abstracts and included/excluded them according to the MIP question. The second author (KH) reviewed the included abstracts. Any disagreements were resolved through discussion.

### Interviews

Six semi-structured interviews with five heads of neonatal departments (out of the total of seven departments in Austria) and one clinical ethicist working in a NICU were conducted with the aim of exploring the choice context specific to Austria. Ethics approval was not requested for interviewing stakeholders based on section 15b, subsection 3a, of the Viennese Law on health institutions.

Interviews were conducted in person or via telephone. All interviews were audio-recorded and afterwards transcribed verbatim. Verbal consent was given by all interview participants prior to recording, audio proof of verbal consent has been collected. An example of the verbatim transcript can be found in the code tree in Table 2.

**Table 2:** Code tree

Overall theme	Code	Sub code	Coding example
Decision-making	Decision models	Guidelines	*“we follow the guidelines ... of the ÖGKJ, uh and uh ... we treat routine- really routinely at 24 + 0, so there’s no question about that if we treat it or not”* (Interviewee 4)
		Grey zone	*“we really try to implement this, this uh new guideline from ... in Austria, yeah. We recognise that in in comparison to the the ... so the guideline in Switzerland and in Germany uh it’s so ... there is a wider uhm ... space ... for for decision making. And and therefore w-we we think we have to offer really a process of uh uh, consultation, counselling in that process. If the mother wants, yeah.”* (Interviewee 6)
		Psychological support	*“the SOP would be that ideally the mother and the father have ... a counselling talk before birth, with the pediatrician and a psychologist. Or let’s say neonatologist and plus psychologist ... it’s not necessarily that both together talk to them, because this is, a resource problem, but both groups have to talk to them, yes. This is the ideal situation.”* (Interviewee 1)
		Ethics committee	*“yeah, we do have, they come together immediately if you need this committee and if. ... the nurses are in this committee, there are people from people different wards who don’t have to do anything with, with the patients, we are in psychologist and so they canmeet immediately and you have a a written m paper afterwards m regarding the discussion and also the decision ...”* (Interviewee 2)
	Communication with parents	Individualized	*“regarding treatment, it’s mostly it’s possible, or always, nearly always to ... work... together with the parents. If you talk to them, if you have enough time for them, if you try to understand them, I think you won’t have a problem, regarding this question.”* (Interviewee 2)
		Paternalism	*“we sometimes really have to fall back and make a paternalistic decision.”* (Interviewee 5)
Ethical challenges	Context	Cultural-religious context	*“nowadays I think or for me is a-, it’s a challenge that we have so many different cultures. ... and ... or we ... maybe ... don’t understand every religious aspect that’s going on in the parents.”* (Interviewee 2)
		Social context (typology of parents and guidelines)	*“So it’s a language problem, and if you look at the immigrants of the last years, it’s not only language, but it’s a s-social situation, they they don’t, they are not really able to imagine the situation (at NICU) ...”* (Interviewee 1)
		Legal context	*“yes it was a challenge before the ethic commission was established. Now we have uh uh a judge in the commission and uh also uh with Medizinrecht, also, uh medical ...”* Interviewee 3)
	Obvious question	Uncertainty (vigorousness assessment)	*“sometimes you are not even sure, i-if is it ih, a 23 weeker, or is it a 24 weeker for instance”* (Interviewee 2)
	Tragic question	Best interest	*“If it were easy to know what the best interest of the child is, we would not need to discuss it”*. (Interviewee 5)
		Moral distress	*“nurses sometimes want to stop therapy. Because of futility and futility is a very difficult thing.”* (Interviewee 1)
		Professional virtues	*“They must have the feeling for the very small and we ... the very ... tiny and and ... also ill babies. So, it’s a, it’s a kind of ... of ‘I like this’. So, at my ... my, my I – I ha- started my trai- aso my training o-on the NICU. First day on the NICU and I went into the NICU and i said ‘Okay, that’s it’.”* (Interviewee 3)

The interview duration ranged from 30 min to 60 min, one single interview lasted 1 h and 40 min. Two researchers conducted and coded the interviews. Interviews were held in English and in some cases, clarifications were phrased in German.

Prior to the data analysis, written transcripts and summaries were sent to the interview participants to confirm the exactness of their quotes. At the time of the HTA’s external review, near to final versions were sent again for final confirmation. If necessary, changes were made in the transcripts and summaries.

To analyse the transcripts, a combination of open coding and structured thematic analysis was applied [12]. This analysis was performed beginning with fragmentation and open-coding of each transcript. Thereby, every fragment received a code such as a word or a short sentence to identify themes.

The main codes and themes were organised in a code-tree. In addition, the themes from the interview topic list served as a structural GL to analyse the interviews. Subsequently, the results of all interviews were edited and common themes and codes integrated. Data analysis was performed using the coding software Atlas.ti (Version 8).

## Results: communication with parents - biases influencing outcomes

The following section is structured in categories of professional biases, parental understanding, and information-giving and choice.

### Professional biases

Communication with parents is shaped by the perceptions and biases of health care professionals in NICU teams. This can, for instance, affect the presentation of treatment options. Parents have to decide based on the content – information provided by the NICU professionals, and the form – the way in which NICU professionals communicate to them. These parental decisions then affect institutional statistics, which in return influence the information provided in the future. Providers need to acknowledge their professional biases, in particular: institutional, personal, and informational bias [13].

#### Institutional bias

There are differences in results of neonates between countries and more importantly between institutions (see Table 3) [14]. One of the explanations comes down to the role of social norms and institutional biases. As suggested by Lantos 2009, “the policy that limits treatment for infants born at 24 weeks of gestation will lead to low survival rates for those infants. The low survival rates will seem to justify and validate the policy, even if the true causal relationship runs in the other direction” [18]. Such a path creates self-fulfilling prophecies because such defaults both reflect on the social norms as well as create them [19].

**Table 3:** Recommendations According to Week of Gestation as of 2015 (German speaking countries update) [14]

Country	Year	Weeks of gestation			
		22	23	24	25
Argentina	2012	CC	NR	NR	NR
Australia	2006	CC	CC	AC	AC
Australia	2013	CC	PW	PW	AC
Austria (Updated according to Austrian GL) [15]	2017	CC	PW	AC	AC
Belgium	2014	CC	CC	PW	PW
Canada	2012	CC	IND	IND	AC
Finland	2014	IND	IND	AC	AC
France	2010	CC	CC	PW	AC
Germany (Updated according to German GL) [16]	2014	IND	PW	AC	AC
FIGO, international association	2006	NR	NR	NR	NR
ILCOR, international association	2006	CC	NR	NR	NR
WAPM, international association	2010	CC	IND	AC	AC
European Resuscitation Council	2010	CC	PW	PW	AC
Ireland	2006	CC	CC	PW	PW
Italy	2008	IND	IND	IND	IND
Japan	2012	NR	NR	NR	NR
Dutch Paediatric Society, the Netherlands	2006	CC	CC	IND	AC
Dutch Ministry of Health, the Netherlands	2010	NR	NR	AC	AC
New Zealand	2011	NR	NR	NR	NR
Poland	2011	CC	CC	IND	AC
Portugal	2012	CC	CC	AC	AC
Singapore	1998	IND	IND	IND	AC
Spain	2004	CC	NR	NR	NR
Sweden	2004	CC	IND	IND	AC
Switzerland (Updated according to Swiss GL) [17]	2011	CC	CC	PW	AC
Nuffield Council, United Kingdom	2006	CC	PW	AC	AC
BAPM, United Kingdom	2009	CC	CC	AC	AC
Royal College of Obstetricians and Gynaecologists, United Kingdom	2014	CC	IND	IND	AC
AAP, United States	2009	IND	IND	IND	IND
ACOG, United States	2012	IND	IND	IND	IND
AHA, United States	2010	CC	PW	PW	AC
Joint Workshop, United States	2014	CC	IND	AC	AC

*AAP* American Academy of Pediatrics, *AC* active care, *ACOG* American College of Obstetricians and Gynecologists, *AHA* American Heart Association, *BAPM* British Association of Perinatal Medicine, *FIGO* International Federation of Gynecology and Obstetrics, *ILCOR* International Liaison Committee on Resuscitation, *IND* individualized care, *CC* comfort care, *NR* no recommendation, *PW* parental wishes, *WAPM* World Association of Perinatal Medicine

With respect to the institutional differences as well as the differences in social norms, Interviewee 5 describes a situation of a Swiss couple delivering an EP infant in Austria. While there is a consensus among all NICUs in Austria that intensive care starts by default in 24th week of gestation and if parents decide so, intensive care is also routinely delivered in 23rd week [15], Interviewee 5 states that in Switzerland and the Netherlands, by default, comfort care is delivered in 23rd week of gestation, because.

*“they are really more conservative… I remember we had a Swiss couple travelling through who did not want to deliver their baby here, but then she had a premature rapture of membranes at 23 weeks plus something…and for her, it was completely normal not to go for this baby. Then we talked a lot with this family and at the end, we convinced them to actively go for this baby and they had a wonderful outcome.”*

The presence of such default bias is also supported by an RCT with adult volunteers that studied the impact of defaults in the NICU context. Participants were randomised to receive either resuscitation or comfort care as the delivery room management default option for a hypothetical delivery of an infant at 23 weeks of GA. Those participants that were told that the default option was resuscitation were more likely to opt for resuscitation and the effect persisted on multivariate regression analysis [20]. The default option created a norm that the participants had the tendency to follow.

#### Personal bias

Not only institutional, but also personal biases may have an impact on the assessment of viability by the NICU professionals and subsequently, on the survival and NDI outcomes. This can be observed on surveys and assessments of hypothetical scenarios, where the variety of conclusions points to the presence of personal attitudes and biases. While NICU professionals in one Australian narrative review underestimated survival and positive outcomes of infants between weeks 22 and 26 of GA [21], in other Australian and US surveys, they also overestimated major neurosensory disability at both week 24 and 28 of GA [22] and long term disability [23]. To the contrary, however, UK NICU professionals in a questionnaire survey overestimated infant survival and underestimated intact infant survival rate [24]. Also, a Finish survey found that NICU professionals with the longest years’ working experience were reluctant to administer steroids to mothers at the lowest weeks of GA to speed up the process of development of the infant [25]. Furthermore, a US study equally revealed the personal biases of the health care professionals by pointing to the correlation between the obstetricians’ willingness to intervene and the periviable outcomes [26]. In this way, personal biases influence the chances for survival of extremely premature infants.

#### Informational bias

NICU professionals make their decisions also based upon their own informational biases. Firstly, reliance on *how the baby looks* right after the delivery is one of the strategies of neonatologists for predicting survival estimates. Same was suggested in the interviews that,

*“if the 23 weeker doesn’t have any vital signs (and the parents don’t want us to do, really everything), comfort care comes in ... the baby shows what to do.” (Interviewee 2)*

However, a possible issue with overreliance on early clinical signs was shown in an Australian study where the neonatologists’ ability to predict survival based on appearance and early response was poor. Videos on ten EP infants were shown to 17 neonatal fellows at 20 s, 2 min, and 5 min after birth. Predictive ability of the neonatal fellows was inaccurate and the level of experience did not affect accuracy of the prediction of survival [27].

Secondly, labelling a periviable infant by the gestational week was shown to skew the estimates for survival and uncover the informational bias of the NICU teams. In a Canadian survey, relying on GA alone led to incorrect assessment of outcomes compared to when the preterm infant was described by its prognosis [28]. Furthermore, in a US survey among obstetrician-gynaecologists, GA was weighted more heavily than parental resuscitation preferences [26], even though the ultrasound evidence of GA may vary by 15% and the gestational weight estimate by 2 weeks [29].

Reliance of on very early ultrasound evidence for GA measurement was also reported in the interviews. Interviewee 5 states that they have confidence in the GA measurement if it comes from the obstetricians within the hospital, because.

*“…with our obstetricians…normally, we know very well the exact date of birth because most of our NICU patients have a very early ultrasound…So, normally, we have very good data there.”*

Furthermore, educational interventions show that personal and informational biases of NICU professionals can improve. A survey examining the relationship between knowledge of participants and their attitude towards resuscitation showed knowledge gaps. After the educational presentation, NICU professionals changed their attitudes and were more prone to resuscitate at all GAs regardless of parental wishes than before [30]. Another survey with hypothetical case scenarios showed that after the educational intervention, respondents improved significantly in the accuracy of their survival and disability estimates [23]. The presence of professional biases – institutional, personal, or informational – thus needs to be acknowledged as it unavoidably influences the survival and NDI outcomes of EP infants in respective institutions.

### Parental understanding

Because preterm infants cannot communicate their preferences autonomously, decisions must be made by proxy [17]. If needed, this surrogate role can be played also by the NICU team or by a societal body such as an ethics committee, or a court of law [31]. In the Austrian NICU decision-making context, ethics committees are in place in all the centres included in the analysis and while some are organized ad hoc by the head of the respective NICU in challenging cases (as stated by Interviewee 5), others have established standard operating procedures that are being followed (as stated by Interviewees 4 and 6). Especially in the *grey zone*, however, it is the legal guardians that, ideally, give consent with the help of NICU professionals in a shared decision-making procedure. Parental decision-making is, however challenging, as Interviewee 4 puts it,

*“I think it’s a real big problem because in this week, the parents must say ‘yes’ or ‘no’ and they must live with this decision.”*

At the same time, the NICU team needs to work in accordance with the parents to the extent possible, as Interviewee 1 suggests,

*“I’m very strongly emphasising this for all our working groups in the NICU that we always have to be in accordance with the parents. If we lose the parents, we lose the infant somehow as well.”*

When communicating with parents, however, one size does not fit all as different parents have different information needs. A recurring theme in the literature calls for parents to be provided with the most accurate prognosis and care options possible in order to make a competent decision [13, 32]. Parents, however, seem to have needs that are so heterogeneous that acting by the principle is not sufficient. As stated by the Interviewee 5 and suggested in the literature, some parents require all the detail possible [13], while others would not be influenced at all by the information provided because of their own value frameworks in place [33].

When making a decision, parents are put under extreme stress [34] and some prefer that the competent NICU professional decides on their behalf [9, 21]. Also, due to the recent dramatic demographic change and the rise in the migrant population, the Austrian NICU professionals face families who have never encountered the idea of *shared decision-making* and so as Interviewee 5 puts it,

*“…we sometimes really have to fall back and make a paternalistic decision.”*

Because parents differ in their capacity and need to understand, it is important that the NICU professionals try to capture the level of understanding of parents and identify their main concerns.

*“We need to develop a sense of who these parents are in order to ensure effective communication for both sides.”* (Interviewee 5)

Interviewee 2 further states that if the communication from the side of NICU professionals is personal and empathetic, it is nearly always possible to work with the parents.

*“If you talk to them, if you have enough time for them, if you try to understand them, I think you won’t have a problem...We answer their questions, we talk about outcome, about survival, about major handicaps, we also talk about what will happen if the baby will come during the next days. If possible, we show them the neonatal intensive care unit...we describe what will happen, that the baby will need respiratory support, tube feeding, central venous line, and so on, and so on…”*

As supported by both the literature and the interviews, the data communicated to the parents need to be personalized because parents have different information needs to be begin with.

#### Real life data and psychological support

There is, however, also a discrepancy between the information that parents can be provided in the NICU and the information that parents would actually need to make a better informed decision. Parents typically receive information about outcomes, prognosis, and care options, however, to make an informed decision, parents would need to know the translation of the numbers they are given into their real life. They would like to find out how the prognosis would influence their family situation, what QoL their child would have, or whether their child would be happy [13].

In case of EP infants, the term QoL is particularly debatable. While there are generic QoL measures (such as 36-Item Short Form (SF-36) or EuroQol five dimension scale (EQ-5D) [35]) and health-related quality of life (HRQoL) measures developed for adults, and older children, there are no established measures for EP infants [36]. There are even family QoL measures in place such as the Family Quality of Life Scale (FQOL) [37]. For EP infants, Boss, Kinsman, and Donohue 2012 suggest that HRQoL metrics on emotional and social functioning for adults and paediatric patients “could be adapted to focus on the role of a neonate in the context of a family unit, as with caregiver ratings of their ability to bond with infant” [36]. Furthermore, they suggest that physical domains such as “pain, energy/neurobehavior, sleep and physical symptoms could be adapted from existing NICU metrics” for instance, the Neonatal Infant Pain Scale, Neonatal Care Unit Network Neurobehavioral Scale, Polysomnography, or Nursing-Child Assessment Feeding Scale [36]. Standardizing the EP infant’s QoL or HRQoL measures could prove beneficial in preventing the neonatal QoL discussion to be narrowed down to analysing only the infant’s physical and cognitive impairments.

Janvier, Barrington, and Farlow 2014 further suggest that parents should be given reassurance about coping strategies, for instance that after experiencing a severe complication, patients tend to return to their baseline QoL after 24 months [13]. Furthermore, they should receive information on the risk of developing psychological problems because there is a substantial increase in depression, anxiety, and financial stress after the birth of a preterm infant, which, however, generally decreases over time [13]. They should also be given information that there is an increase in family cohesion, less conflict than in typical families, and no increase in divorce in families with preterm infants [13].

The content of psychological support was not discussed in the interviews, but with respect to psychological support for families with EP infants, the Interviewees reported a homogenous use across all five perinatal care centres. All centres reported that there was a psychological support for parents. In the centre of Interviewee 5, one psychologist supported the family through the entire hospital stay, whereas in the centre of Interviewee 1, the family was transferred from the obstetrics psychologist to the NICU psychologist. Furthermore, Interviewee 3 suggested that psychological support ideally comes in before birth, when both, a neonatologist as well as a psychologist talk to the parents about the possible courses of action. However, Interviewee 5 reminded that counselling also comes from nurses, who spend a lot of time with parents at the bedside.

Because parents come from different backgrounds and have different information needs, the communication from the side of NICU professionals needs to adjust. Moreover, apart from medical data about outcomes, psychological support and real life psychological data about the impact of having an EP infant need to be communicated to the parents [13].

### Information-giving and choice biases

When communicating with parents, no neutral or uniformed tools can solve the problem of the biases of NICU professionals and the individuality of parents. For that reason, doctors, counsellors, as well as nurses need to be aware of their own biases and they need to use their best judgment to provide balanced information to parents that is also personalized. As the Interviewee 5 puts it that is their NICU,

*“parents get an idea of the medical data as well as an idea of what the NICU team thinks is worth-while doing (not with regards to resources and money, but in the interest of the EP infant and the family).”*

In the process of passing the information to parents, however, different cognitive biases are at play (see Table 4), in particular, a framing effect.

**Table 4:** Cognitive biases and their influence on decision-making [13]

Cognitive biases	The possible influence of the bias on the communication between NICU professionals and parents
*Anchoring effect:* tendency to rely on the first piece of information received (the anchor). This piece of information is used to make subsequent judgments.	Speaking about risks before benefits may create a negative anchor on parents’ perception.
*Focusing effect:* placing too much importance on one aspect of the situation that falsifies the prediction of a future outcome.	Speaking about all possible disabilities an EP infant may have for a lengthy period and not speaking about the likely abilities.
*Availability effect:* estimation of a probability of an event that is associated with vivid memories of similar events happening before.	If doctors tell the parents that their child is going to die three times, but it survives nonetheless, parents overestimate the chances for survival in case of another event.
*Effective forecasting:* individuals often predict the future health states inaccurately. Individuals tend to be more resilient than they predict.	Parents may find it difficult to imagine living with a disabled child, but manage it better than they anticipated nonetheless.
*Loss aversion*: tendency to strongly prefer avoiding losses to acquiring gains (the loss of 100 EURO causes more loss of satisfaction that the satisfaction gained from winning 100 EURO).	Framing the information via losses and gains may have an impact on parents, i.e., losing a child vs getting a child.

#### Framing effect

Framing effect is one of many cognitive biases that reveals that people react to a particular choice in different ways depending on the way it is presented. Sometimes, there seems to be a discrepancy between what NICU professionals think that they communicate to parents and what parents actually perceive. Structured interviews with mothers and counsellors revealed that mothers perceived the counselling about resuscitation of extremely premature infants directive, even though the majority of counsellors believed that mothers were given a choice of treatment options [38].

When communicating with parents, framing effect of how the data is presented is inevitable. Communicating proportional outcomes constitutes the majority of information that is being given to parents, however, many individuals do not understand percentages [13]. Patients tend to choose a procedure where the risk of death is described as 24 out of 100, but they tend not to choose the one where the risk is described as 120 out of 1000. Even though the risk is smaller in the latter, patients presumably tend not to choose that procedure because 120 is a larger number than 24 [13].

The question of framing effect did not come up in any of the interviews, but for the sake of better communication with parents, an instant translating system called Videodolmotsch that interacts with a real interpreter via a screen was used in the centre of Interviewees 5 and 6.

*“,,,we always discuss with parents, of course, which is difficult if you are confronted with families who do not have the cultural, religious, or intellectual basis to decide. We still discuss it with them. We have a Videodolmetsch system where we at least try to show those parents, or pregnant women, what the odds of complications and NDIs are. But normally, it is like “Please do everything for our baby”.”* (Interviewee 5)

The attitude of parents to save the EP infant at all costs is, as shown above, context specific as it is subject to the respective societal norms. The impact of framing seems to persist nonetheless as a randomized survey found a trend towards a framing effect on the treatment preference in NICU decision-making. Participants for whom the prognosis was framed as survival and non-disability rates were more likely to choose resuscitation than participants for whom prognosis was framed as mortality and disability rates [39]. Framing effect can also be seen when presenting a list of complications of an intervention, although there is also evidence against it [40]. When the list starts with the rarest and ends with them most common complication, patients tend to choose against the intervention. When presented in the opposite order, patients tend to choose for it [13]. Other cognitive biases that have an impact on the decision-making at the *limit of viability* are described in Table 4.

## Discussion

This literature review outlined the current evidence on decision-making practices, especially on neonatologist’s communication strategies at the *limit of viability*. Data from the systematic literature search were supported by specific data from the interviews with Austrian heads of NICUs and a clinical ethicist. The overlap between the literature reviewed and the content of the semi-structured interviews is the main advance that this paper adds on the existing literature. The categories of biases found in the literature were also found to be present in the Austrian neonatal context, namely professional biases, parental understanding, and the process of information giving were identified as aspects possibly influencing survival and NDI outcomes.

### Outcomes and biases

Especially within the *grey zone*, the difference in practices may lead to a difference in outcomes. The Swiss policy of routinely providing comfort care at 23 weeks of GA leads to low survival rates for those infants [17]. These outcomes then become part of institutional statistics that further influence counselling and so the outcomes based on historical data influence the present decision-making of parents and thus the future outcomes. Institutional biases such as this and other biases that are at play during shared decision-making processes (see Table 4) are understood to be in part responsible for the variation in outcomes between hospitals.

As behavioural sciences argue, the real decision-making involves the use of mental shortcuts (biases) and one ought not to perceive them as undesirable interferences with the rational decision-making process [41]. It is necessary to recognize the impact of these biases on the decision-making in NICUs as they are an inevitable part of the process that needs to be taken into account when developing GLs for shared decision-making procedures [13]. Good practice decision models need to include not only psychological support for the parents and ethics support for the NICU professionals that are already to some extent acknowledged, but also support for neonatologists in terms of communication strategies with parents as those may influence outcomes as well [42]. It is argued here that there is a need for trainings of NICU professionals aiming first at recognition of the biases and second, at the reduction of influence of their personal and informational biases on the parents.

### Limitations

In terms of limitations to this literature review, we acknowledge, that firstly, we limited the search language to German and English, which presumably led to leaving out of some literature. Nonetheless, we consider the literature that we used to be robust enough to provide a good overview of the decision-making models in place.

Secondly, even though this literature review was based on a systematic literature search complemented by a thorough hand search, the way of reporting remained non-systematic because we could not find a common denominator that would allow for comparison between the variety of studies. Hence, also no quality assessment or risk of bias tools were used.

## Conclusion

Communication strategies of choice options to parents may have an impact on the way parents decide and hence also on the survival and NDI outcomes of EP infants. Professional biases, parental understanding, and the process of information giving were identified as aspects possibly influencing outcomes. For these reasons, it is important to address the impact of communication in the management GLs of EP infants as well as in in-house trainings for NICU professionals.

## Data Availability

Search strategies as well as all data generated or analyzed during this study are included in this published report: Stanak M, Hawlik K. Perinatal Care at the threshold of viability: Decision-making at the threshold of viability and ethical challenges at Neonatal Intensive Care Units (NICUs). LBI-HTA Project No.: 97b; 2017. Wien: Ludwig Boltzmann Institute for Health Technology Assessment.

## Abbreviations

CRD: Centre for Reviews and Dissemination
EP: Extremely preterm
EQ-5D: EuroQol five dimension scale
GA: Gestational age
GL: Guideline
HRQoL: Health-related quality of life
HTA: Health technology assessment
MIP: Methodology, issue, participants
NDI: Neurodevelopmental impairment
NICU: Neonatal intensive care unit
QoL: Quality of life
SF-36: Medical outcomes study 36-item short form

## References

[1] Glass HC, Costarino AT, Stayer SA, Brett CM, Cladis F, Davis PJ (2015). Outcomes for Extremely Premature Infants. Anesth Analg.

[2] Statistik der natürlichen Bevölkerungsbewegung [database on the]. 2016. Available from: http://www.statistik.at/web_de/statistiken/menschen_und_gesellschaft/bevoelkerung/geborene/medizinische_und_sozialmedizinische_merkmale/index.html. [cited 25/10/2017]

[3] Patel RM (2016). Short- and long-term outcomes for extremely preterm infants. Am J Perinatol.

[4] Jarjour IT (2015). Neurodevelopmental outcome after extreme prematurity: a review of the literature. Pediatr Neurol.

[5] Catlin A (2015). Palliative and end-of-life Care for Newborns and Infants. Adv Neonatal Care.

[6] Mercurio MR. Periviable birth (limit of viability). UpToDate Inc; 2017; Available from: https://www.uptodate.com/contents/periviable-birth-limit-of-viability. [cited 09/11/2017]

[7] Zeitlin J, Szamotulska K, Drewniak N, Mohangoo A, Chalmers J, Sakkeus L (2013). Preterm birth time trends in Europe: a study of 19 countries. BJOG Int J Obstet Gynaecol.

[8] Myrhaug HTBK, Hov L, Håvelsrud K, Reinar LM. Prognose for og oppfølging av ekstremt premature barn: En systematisk oversikt. Oslo: Norwegian Institute for Public Health; 2017.

[9] Sunstein CR. Nuding Health: Health Law and Behavioural economics,. Cohen G, Fernandez. Lynch H, Robertson CT, editors. Baltimore: John Hopkins University Press; 2016.

[10] Stanak Michal (2018). Professional ethics: the case of neonatology. Medicine, Health Care and Philosophy.

[11] Stanak M, Hawlik K (2017). Perinatal care at the threshold of viability part II: decision-making at the threshold of viability and ethical challenges at neonatal intensive care units (NICUs).

[12] CT DMA (2014). Principles of social research.

[13] Janvier A, Barrington K, Farlow B (2014). Communication with parents concerning withholding or withdrawing of life-sustaining interventions in neonatology. Semin Perinatol.

[14] Guillén Ú (2015). Guidelines for the Management of Extremely Premature Deliveries: a systematic review. Pediatrics..

[15] Berger A (2017). Erstversorgung von Frühgeborenen an der Grenze der Lebensfähigkeit. Monatsschr Kinderheilkd.

[16] Deutschen Gesellschaft für Gynäkologie und Geburtshilfe, Deutschen Gesellschaft für Kinder- und Jugendmedizin, Deutschen Gesellschaft für Perinatale Medizin, Akademie für Ethik in der Medizin, Gesellschaft für Neonatologie und Pädiatrische Intensivmedizin Frühgeborene an der Grenze der Lebensfähigkeit 2014.

[17] Berger TM, et al. Perinatal care at the limit of viability between 22 and 26 completed weeks of gestation in Switzerland. Swiss Med Wkly. 2011;141:w13280. 10.4414/smw.2011.13280 22009720

[18] Lantos J, Meadow W (2009). Variation in treatment of infants born at the borderline of viability. Pediatrics..

[19] Rysavy MALL, Bell EF, Das A, Hintz SR, Stoll BJ (2015). Between-hospital variation in treatment and outcomes in extremely preterm infants. N Engl J Med.

[20] Haward MF, Murphy RO, Lorenz JM (2012). Default options and neonatal resuscitation decisions. J Med Ethics.

[21] Ireland S, Ray R, Larkins S, Woodward L (2015). Factors influencing the care provided for periviable babies in Australia: a narrative review. Reprod Health.

[22] Boland RA, Davis PG, Dawson JA, Doyle LW (2016). What are we telling the parents of extremely preterm babies?. Aust NZ J Obstet Gynaecol.

[23] Blanco F, Suresh G, Howard D, Soll RF (2005). Ensuring accurate knowledge of prematurity outcomes for prenatal counseling. Pediatrics..

[24] Chan KL, Kean LJ, Marlow N (2006). Staff views on the management of the extremely pretern infant. Eur J Obstet Gynecol Reprod Biol.

[25] Taittonen L, Korhonen PH, Palomaki O, Luukkaala T, Tammela O (2013). Opinions of the counselling, care and outcome of extremely premature birth among healthcare professionals in Finland. Acta Paediatr.

[26] Tucker Edmonds B, McKenzie F, Farrow V, Raglan G, Schulkin J (2015). A national survey of obstetricians’ attitudes toward and practice of periviable intervention. J Perinatol.

[27] Brett JM (2010). Clinical assessment of extremely premature infants in the delivery room is a poor predictor of survival. Pediatrics..

[28] Dempsey EM, Barrington KJ (2006). Diagnostic criteria and therapeutic interventions for the hypotensive very low birth weight infant. J Perinatol.

[29] Kett JC (2015). Prenatal consultation for extremely preterm neonates: ethical pitfalls and proposed solutions. J Clin Ethics.

[30] Doucette S, Lemyre B, Daboval T, Dunn S, Akiki S, Barrowman N (2017). Effect of an educational presentation about extremely preterm infants on knowledge and attitudes of Health care providers. Am J Perinatol.

[31] Wallner J (2009). Careful planning of the decision-making process in neonatology: ethical orientation. Z Geburtsh Neonatol.

[32] Batton DG (2009). Committee on F, newborn. Clinical report--antenatal counseling regarding resuscitation at an extremely low gestational age. Pediatrics..

[33] Boss RD, Hutton N, Sulpar LJ, West AM, Donohue PK (2008). Values parents apply to decision-making regarding delivery room resuscitation for high-risk newborns. Pediatrics..

[34] Gallagher K, Martin J, Keller M, Marlow N (2014). European variation in decision-making and parental involvement during preterm birth. Arch Dis Child Fetal Neonatal Ed.

[35] Coons SJ, Rao S, Keininger DL, Hays RD (2000). A comparative review of generic quality-of-life instruments. PharmacoEconomics..

[36] Boss RD, Kinsman HI, Donohue PK (2012). Health-related quality of life for infants in the neonatal intensive care unit. J Perinatol.

[37] Hoffman L, Marquis J, Poston D, Summers JA, Turnbull A (2006). Assessing family outcomes: psychometric evaluation of the beach center family quality of life scale. J Marriage Fam.

[38] Keenan HT, Doron MW, Seyda BA (2005). Comparison of mothers’ and counselors’ perceptions of predelivery counseling for extremely premature infants. Pediatrics..

[39] Haward MF, Murphy RO, Lorenz JM (2008). Message framing and perinatal decisions. Pediatrics..

[40] Haward MF, John LK, Lorenz JM, Fischoff B (2012). Effects of description of options on perantal perinatal decision-making. Pediatrics..

[41] Halpern D (2015). Inside the nudge unit.

[42] Berger TM (2015). Guidelines for the management of extremely preterm deliveries in the grey zone of viability between 23 and 24 weeks’ gestation vary widely in developed countries. Evid Based Med.

